# Editorial: Parvoviruses: from basic research to biomedical and biotechnological applications

**DOI:** 10.3389/fmicb.2023.1194926

**Published:** 2023-05-15

**Authors:** Mario Mietzsch, Jianming Qiu, José M. Almendral, Maria Söderlund-Venermo

**Affiliations:** ^1^Department of Biochemistry and Molecular Biology, University of Florida, Gainesville, FL, United States; ^2^Department of Microbiology, Molecular Genetics and Immunology, University of Kansas Medical Center, Kansas City, KS, United States; ^3^Centro de Biología Molecular Severo Ochoa, Departamento de Biología Molecular, Universidad Autónoma de Madrid, Madrid, Spain; ^4^Department of Virology, University of Helsinki, Helsinki, Finland

**Keywords:** parvovirus B19, human bocavirus, adeno-associated virus, minute virus of mice, feline chaphamaparvovirus, parvovirus

Parvoviruses are small non-enveloped icosahedral viruses with linear single-stranded DNA genomes of 4–6 kb with terminal repeats forming hairpin structures at both ends. To date, the family *Parvoviridae* comprises three subfamilies; *Parvovirinae, Densovirinae*, and *Hamaparvovirinae*, with virus members infecting either vertebrate or invertebrate hosts, or both, respectively (Pénzes et al., 2020). Parvovirus replication is highly dependent on host cell factors, including DNA polymerases. Their replication mostly takes place in actively dividing cells but sometimes also in quiescent cells. Parvoviruses cause subclinical to deadly infections and often persist in the host with unknown consequences. Some parvoviruses, like H-1, are used as oncolytic viruses for cancer treatment, and others as vectors for gene therapy, like the adeno-associated viruses (AAVs) (Angelova et al., 2021; Pupo et al., 2022). The most widely known parvovirus pathogens of humans are parvovirus B19 (B19V) and human bocavirus 1 (HBoV1) (Qiu et al., 2017), and of animals, e.g., the canine, mink, bovine, and porcine parvoviruses, for which vaccines are available, and minute virus of mice (MVM), which has been most widely studied (Figure 1; Jager et al., 2021). Many densoviruses are highly pathogenic for insects or crustaceans, and some are being used for pest control (Johnson and Rasgon, 2018).

**Figure 1:**
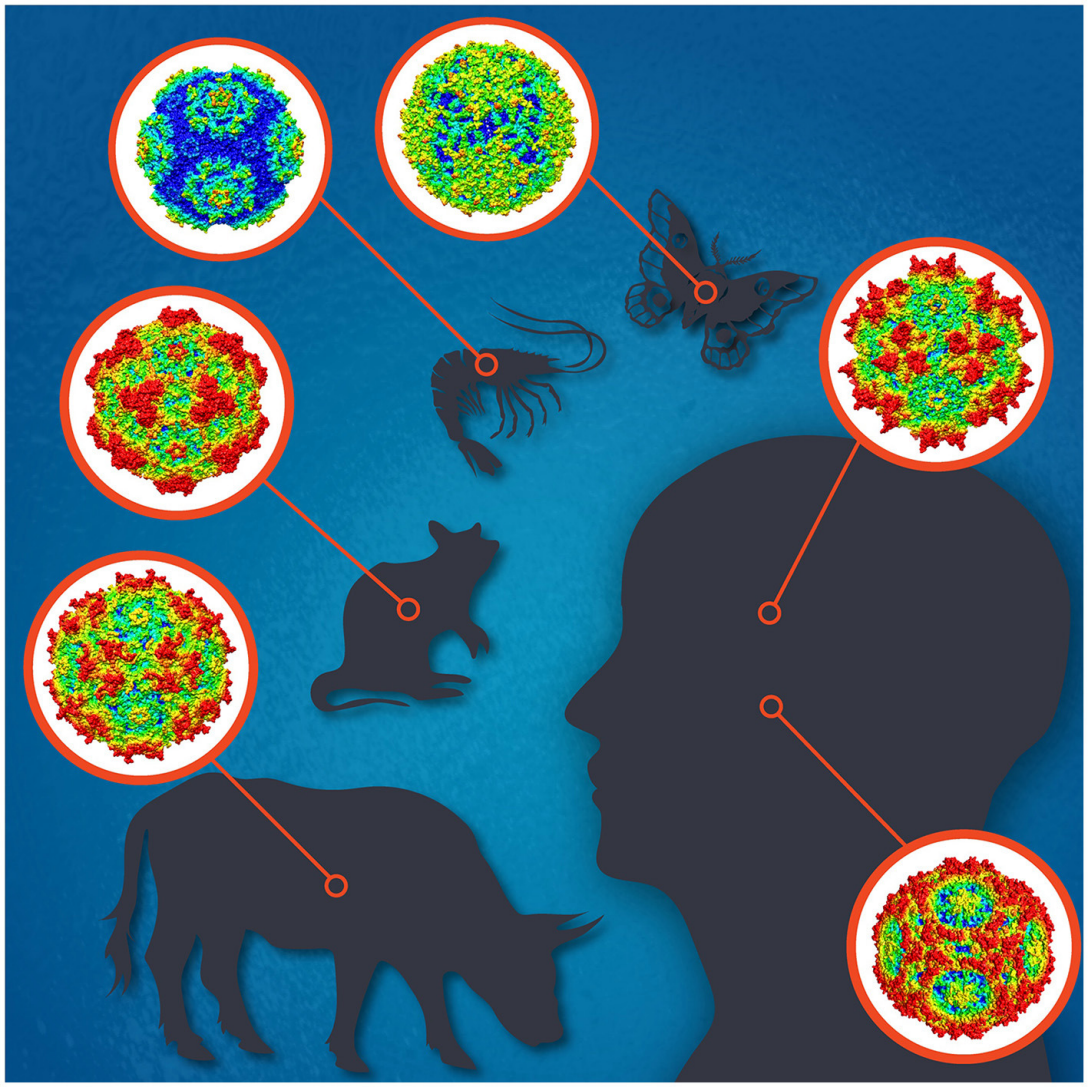
The icosahedral capsids for selected parvoviruses of human [adeno-associated virus 2 **(top)**, parvovirus B19 **(bottom)**], cow (bovine parvovirus), mouse (minute virus of mice), shrimp (Penaeus monodon metallodensovirus), and moth (Galleria mellonella densovirus) are shown. Illustration designed by Jane Hsi.

This Research Topic focuses on the recent advances across the entire spectrum of molecular, clinical, and applied parvovirus research.

In the topic of parvovirus — cell-surface interaction, Large and Chapman revisited the previously reported capsid-receptor complex structures for the AAVs and compared their binding sites to the epitopes of neutralizing antibodies. The authors suggested that the proteinaceous AAV Receptor (AAVR), KIAA0319L, is one of the main contributors to cellular transduction, while the cell surface glycans should be demoted to attachment factors, and the identified co-receptors only have minor accessory roles. They concluded that antibody interference with AAVR binding might be a more prevalent mechanism for AAV neutralization rather than the interference with glycan attachment.

The role of parvovirus-glycan interaction in the infection may, however, differ between parvovirus systems. For instance, surface glycans, as functional receptors, driving virion entry was reported by Calvo-López et al., while retargeting the protoparvovirus MVM to the tumor vasculature with vascular endothelial growth factor (VEGF) blocking peptides inserted at the sialic acid (sia) binding domain near the 2-fold axis. The MVM-VEGF chimeric virion showed altered tropism determined by capsid contacts with cell-type specific sia glycans driving the virion to the endosome, where a drastic structural transition onsets productive entry. In addition, the sia-dependent entry could be further enhanced by controlled neuraminidase treatments. The identification of the intracellular traffic to the endosome as a key step of MVM entry may help understanding the tropism and host range of other parvoviruses.

Other topics of interest addressed the interaction of a pathogenic parvovirus with mammalian hosts in nature. By modern metagenomics, many novel emerging parvoviruses have been discovered, like the feline chaphamaparvovirus (FeChPV) in 2019 (Li et al., 2020). Hao et al. reported pathogenic features of an outbreak of FeChPV in China. They showed severe symptoms in the upper respiratory tract, severe lymphadenitis and encephalitis corresponding to the presence of viral DNA in the feline tissues. Interestingly, viral protein (VP) 1 showed seven and the non-structural protein 1 eight unique amino-acid mutations, the roles of which, in the pathogenicity of the virus, deserve further research.

Human parvovirus B19 was long the only known human-pathogenic parvovirus, causing erythema infectiosum, arthropathies, anemias, and fetal death (Qiu et al., 2017), whereas HBoV1, causing pediatric mild to life-threatening acute respiratory-tract infections (ARTI), was discovered in 2005 (Allander et al., 2005; Christensen et al., 2019). Xu et al. searched for parvoviral DNA in a total of 427 intestinal biopsy specimens, as paired diseased and healthy mucosa. Only three (1.6%) individuals exhibited intestinal HBoV DNA, compared to 50, 47, 31, and 27% of patients with malignancy, ulcerative colitis, or adenomas, and in healthy subjects, respectively, who harbored B19V DNA. B19V DNA persisted mostly in the mucosal B cells of lymphoid follicles and in vascular endothelial cells. When comparing B19V DNA-positive and -negative healthy ileum biopsy specimens, RNA sequencing identified 272 differentially expressed cellular genes, which activated intestinal cell viability and inhibited apoptosis. B19V-DNA persistence was thus shown to modulate host gene expression, with yet unknown clinical outcomes.

There were two studies of HBoV1 from China. De et al. screened respiratory specimens from children with ARTI by three methods. Out of 9,899 airway samples, 681 (7%) were positive for HBoV1 DNA by a capillary electrophoresis-based multiplex PCR (CEMP) assay, 37/47 samples tested exhibited HBoV1-VP3 antigen by indirect-immunofluorescence assay (IFA), and HBoV1-specific IgG was tested by IFA for four patients with available paired sera. They concluded that the combinatorial results of DNA, antigen, and serology tests are proof of HBoV1 being a genuine pathogen for ARTI in children. Wang et al. evaluated the prevalence, epidemiology, and clinical characteristics of HBoV among children with ARTI. They screened nasopharyngeal swabs for 16 viral and 16 bacterial pathogens by two multiplex PCRs, and compared clinical parameters in various groups. HBoV1 was the most common virus (56/199, 28.1%) and the second most common pathogen detected, after *S. pneumoniae* (71/199, 35.7%). Forty-two (75%) cases were HBoV1 single-virus infections and 14 (25%) were HBoV single-pathogen infections. Vomiting or diarrhea was detected in five children in the sole-HBoV group vs. none in the coinfection group, but the latter group exhibited a higher proportion of wheezing.

In parvovirus gene therapy approaches, Shoti et al. updated the manufacturing protocol of a novel no-end (NE) AAV DNA that consists of a gene expression cassette covalently flanked by AAV2 inverted terminal repeats (ITRs), and assessed various NE-AAV DNA forms in immortalized hepatocyte cell lines by transfection. Finally, they developed an NE-AAV DNA that has a human Factor IX (hF.IX) gene expression cassette under the control of a human liver-specific transthyretin promoter, and proved it to express, in human hepatocyte cells at four-weeks post-transfection, ~6-fold higher levels of hF.IX than that from a linear TTR-hF.IX DNA construct. This novel NE AAV DNA opens another venue for gene therapy of hemophilia in children.

## Author contributions

All authors listed have made a substantial, direct, and intellectual contribution to the work and approved it for publication.
